# A Joint Low-Rank and Sparse Method for Reference Signal Purification in DTMB-Based Passive Bistatic Radar

**DOI:** 10.3390/s21113607

**Published:** 2021-05-22

**Authors:** Luo Zuo, Jun Wang, Te Zhao, Zuhan Cheng

**Affiliations:** National Laboratory of Radar Signal Processing, Xidian University, Xi’an 710071, China; zuoluo@stu.xidian.edu.cn (L.Z.); tezhao@stu.xidian.edu.cn (T.Z.); cheng@stu.xidian.edu.cn (Z.C.)

**Keywords:** passive bistatic radar (PBR), compressive sensing (CS), signal reconstruction, low-observable target

## Abstract

In a digital terrestrial multimedia broadcasting (DTMB)-based passive bistatic radar (PBR) system, the received reference signal often suffers from serious multipath effect, which decreases the detection ability of low-observable targets in urban environments. In order to improve the target detection performance, a novel reference signal purification method based on the low-rank and sparse feature is proposed in this paper. Specifically, this method firstly performs synchronization operations to the received reference signal and thus obtains the corresponding pseudo-noise (PN) sequences. Then, by innovatively exploiting the inherent low-rank structure of DTMB signals, the noise component in PN sequences is reduced. After that, a temporal correlation (TC)-based adaptive orthogonal matching pursuit (OMP) method, i.e., TC-AOMP, is performed to acquire the reliable channel estimation, whereby the previous noise-reduced PN sequences and a new halting criterion are utilized to improve channel estimation accuracy. Finally, the purification reference signal is obtained via equalization operation. The advantage of the proposed method is that it can obtain superior channel estimation performance and is more efficient compared to existing methods. Numerical and experimental results collected from the DTMB-based PBR system are presented to demonstrate the effectiveness of the proposed method.

## 1. Introduction

PBR systems have been extensively studied for the last several decades due to the several advantages they offer over active radars. Since PBRs do not require an additional frequency channel, they are virtually undetectable to surveillance receivers [1]. Further, PBRs have superior low-altitude coverage capability. PBR exploits available non-cooperative transmitters as its illuminators of opportunity, such as frequency modulation (FM) radio [2], digital audio broadcasting (DAB) [3,4], digital video broadcasting (DVB) [5,6], global navigation satellite system (GNSS) [7,8], digital terrestrial multimedia broadcasting (DTMB) [9,10], etc. Among these illuminators of opportunity, DTMB has attracted much attention for its high range resolution and wide coverage area.

DTMB is a digital television terrestrial broadcasting (DTTB) international standard developed by China, which has been widespread in Chinese towns and cities. Further, the power of DTMB is large (1–5 kW) and stable. Therefore, it has the natural advantage of network cooperative and far distance detection. The DTMB adopts the time-domain synchronous orthogonal frequency division multiplexing (TDS-OFDM) as the baseline modulation technology [11]. TDS-OFDM uses three pseudo-noise (PN) sequences padding modes as a guard interval (GI) as well training sequences for both single and multi-carrier block transmissions, which is an advantage for synchronization and channel estimation [12]. With the better range resolution, of about 40 m, DTMB enables improved target localization compared with GSM and FM [13]. Moreover, DTMB is tilted towards the ground, therefore DTMB-based PBR has the natural advantage of low-altitude detection characteristics, such as UAV and drone detection.

In PBR systems, target detection is committed by calculation of cross-ambiguity function (CAF) of the surveillance and reference signal [14]. The surveillance signal is collected by an antenna directed towards a specific area of interest. In addition to target returns, the surveillance signal also inevitably includes the direct illumination signal originating from the transmitter, reflections from other objects, such as ground, hills, and buildings, which are called clutter, and noise [15]. Since the sidelobes of those clutters can completely mask target returns, adaptive filtering is employed to remove unwanted components (clutter) before the application of CAF [16,17]. In the processing scheme of adaptive filtering and CAF, the reference signal plays a crucial role. In theory, the reference signal is a perfect replica of the direct illumination signal. Even though PBR systems do not have a direct access to the direct illumination signal, they can obtain the reference signal directly from the transmitter by using a directional antenna towards the transmitter [18,19]. However, the received signal is always noisy and multipath clutter-polluted, which results in clutter remaining in the adaptive filtering process, and therefore leads to deterioration of the target detection performance [20]. Particularly, for the low-observable target, such as unmanned aerial vehicle (UAV) detection, the impact of impure reference signal is more serious because of the low radar cross-section (RCS) [21]. Therefore, to obtain the desired target detection ability in DTMB-based PBR, the reference signal should be as pure as possible.

A popular approach is to reconstruct the direct illumination signal by demodulating the impure reference signal to the bit level and then modulating it again, and therefore a nearly perfect replica of the direct DTMB signal is obtained [22,23]. In this approach, channel impulse response (CIR) estimation and equalization play a crucial role in reference signal reconstruction, which is used to eliminate the multipath effect. Generally, CIR estimation develops in three directions. The first one exploits the good autocorrelation characteristics of PN sequence, such as iterative subtraction [24]. In real PBR systems, however, the system sampling rate is not an integer multiple of the baseband symbol rate *T*_s_ (non-*T*_s_-spaced), because of hardware resource constraint [25]. Non-*T*_s_-spaced will induce a reduction in autocorrelation characteristics, and therefore worsen the CIR estimation performance. Hence, symbol synchronization should be performed before the PN correlation. However, its CIR estimation still contains many false estimations due to the mutual interference between channels and the influence of noise. The second direction is based on the least-squares (LS) criterion, such as the least mean square (LMS), recursive least square (RLS) algorithm [26,27]. Since the LS algorithm is based on the dense distribution of CIRs, a large number of training sequences are required to estimate CIRs. This means LS will suffer from high calculation costs and is more susceptible to noise. The third is a CS-based signal recovery algorithm, such as the orthogonal matching pursuit (OMP) algorithm, aims to estimate the CIR due to the practical wireless channel being sparse in nature [28,29]. However, the scheme suffers from noise effect and it usually adopts the fixed number of iterations, which decreases the CIR estimation accuracy. Moreover, this popular reconstruction approach increases the complexity of the PBR signal processing and is not practical due to the 170/510 frames delay caused by de-interleaving and forward error correction (FEC) decoding.

To tackle the problems in the aforementioned method, the received reference signal model with the multipath effect is constructed and a novel signal purification method based on low-rank and sparse features is proposed. More specifically, synchronizations, including symbol, carrier, and sampling rate synchronization, are performed to reference signal at first, and the corresponding PN sequences can be obtained. After that, we innovatively exploit the inherent low-rank structure of DTMB signals and utilize the singular value decomposition (SVD) algorithm to suppress the noise in PN sequences. Furthermore, the temporal correlation (TC)-based adaptive OMP method, i.e., TC-AOMP, is proposed to estimate the sparse CIR, in which the previous noise-free PN sequences and a new halting criterion defined based on TC result are utilized to further improve the channel estimation accuracy. In the end, the purification reference signal is obtained via equalization operation. Generally speaking, the proposed method has several attractive advantages. Firstly, the proposed method exploits the low rank of DTMB signal and sparse feature of the practical channels, which significantly decreases the false alarm of the CIR estimation compared with PN correlation and RLS methods. Secondly, unlike the conventional OMP signal recovery method, the proposed method leverages the low-rank structure of DTMB signal for noise reduction and the halting criterion is determined adaptively, which greatly enhances the CIR estimation accuracy. Eventually, the effectiveness of the proposed method is verified using numerical data and measured data from an experimental DTMB-based PBR system.

The rest of the paper is organized as follows. The signal model is established and analyzed in Section 2. Relevant synchronization operations are introduced in Section 3. A joint low-rank and sparse method for reference signal purification is proposed in Section 4. In Section 5 and Section 6, the effectiveness of the proposed method is demonstrated using simulated and measured data, respectively. Relevant summary and conclusions are given in Section 7.

## 2. System Model of DTMB-Based PBR

**Figure 1:**
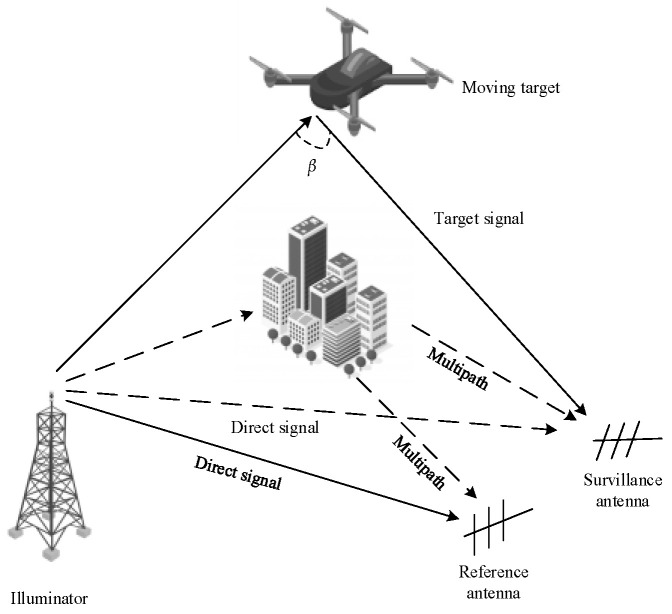
PBR system model.

Figure 1 shows the observation model of PBR. The DTMB transmitter is an omni-directional antenna in azimuth angle. The PBR receiver collects both a reference signal via a line-of-sight (LOS) path direct from the transmitter and a surveillance signal reflected from target of interest. Besides this, several attenuated replicas of the direct signal at short delay because of static multipath (clutter) are introduced at the receiver. *β* denotes the bistatic angle.

### 2.1. Frame Structure of DTMB Signal

**Figure 2:**
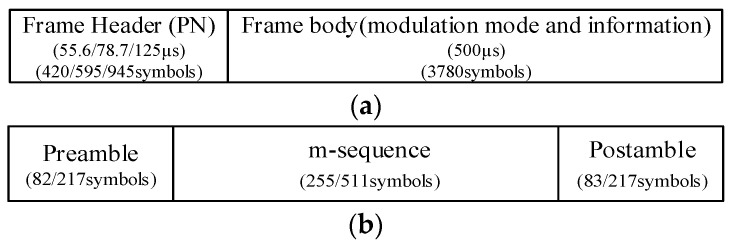
DTMB signal: (**a**) frame structure; (**b**) frame header structure of PN420 and PN945.

The DTMB adopts the TDS-OFDM as the baseline modulation technology. In the physical layer transmission of the DTMB standard, a signal frame consists of frame header (FH) and frame body (FB), as presented in Figure 2a [30]. The PN sequence, namely FH, is padded as the guard interval (GI) to FB for either multi- or single-carrier block transmission. The PN padding structure can not only contribute to the frame synchronization but also improve the spectrum efficiency due to the perfect knowledge of PN sequence that can be obtained in advance. The baseband symbol rate *T*_s_ for both FH and FB is 7.56 MS/s.

In FH, three types of PN sequences, PN420, PN595, and PN945, are defined. The FB consists of a preamble, an m-sequence, and a postamble in the case of PN420 or PN945 is used in a signal frame. The m-sequence is generated with a Fibonacci-type linear feedback shift register (LFSR), while its cyclic extensions constitute preamble and postamble. Figure 2b shows the FH structure. In FB, there are two kinds of options on the number of subcarrier *C*, *C* = 1 for single-carrier (the corresponding FH is PN595) and *C* = 3780 for multi-carrier mode (the corresponding FH is PN420 or PN945). Without loss of generality, only multi-carrier mode is analyzed in this paper.

The transmitted signal that includes one TDS-OFDM symbol, i.e., one signal frame, is given as

(1) $$g(t)=\left\{ \begin{array}{ll} pn & (m-1)T_{e}<t\leq (m-1)T_{e}+T_{p}\\ s(t) & (m-1)T_{e}+T_{p}<t\leq mT_{e} \end{array}\right.$$

where *T*_e_ is the duration of one signal frame; *T*_p_ is the duration of the FH, i.e., the PN sequence *pn*; *m* = 1,2,…, *M* denotes the frame index, *M* is the number of the frame in the transmitted signal. *s*(*t*) is the OFDM data block, i.e., the frame body signal, which is expressed as

(2) $$s(t)=\sum_{c=0}^{C-1}a_{c}\exp(j2\pi c\Delta ft)$$

where *c* indexes the carriers, *C* is the total carriers; *a_c_* is the complex modulation value for carrier *c*; Δ*f* is the subcarrier frequency spacing.

### 2.2. Signal Model

In this section, a reference signal model with noise and multipath clutter is established. We consider the multipath fading channel as an *L*_tap_-order FIR filter and enough length of FH to mitigate the multipath effect. Therefore, the interference between two adjacent OFDM data blocks can be avoided in the DTMB system [31]. Since the location of the transmitter and receiver are fixed in the PBR system, we assume that CIR does not vary within one signal frame. The CIR can be expressed as

(3) $$h(t,\tau )=\sum_{l=0}^{L_{\mathrm{tap}}-1}h_{l}\delta (t-\tau_{l})$$

where *L*_tap_ denotes the multipath channel taps number, and *h_l_* and *τ_l_* are the complex amplitude and time delay of the *l*th tap in the CIR. It is assumed that the CIR in each path is independent.

After passing through the multipath channel, the received reference signal can be represented as a linear convolution between the transmitted signal and the CIR, as

(4) r(t)=g(t)∗h(t,τ)+n(t)

where (*) denotes the convolution operation; *n*(*t*) is the additional white Gaussian noise.

In practice, we work with sampled signals, and for simpler analysis, a discrete single frame signal is considered. Therefore, the received signal can be represented as

(5) $$r_{m}=g_{m}*h_{m}+n_{m}$$

where $g_{m}={\left[g_{m}[0],g_{m}[1],\cdots ,g_{m}[L-1]\right]}^{T}$, which consists of FH, i.e., PN sequences $pn={\left[pn[0],pn[1],\cdots ,pn[L_{pn}-1]\right]}^{T}$ and OFDM data block $s_{m}={\left[s_{m}[0],s_{m}[1],\cdots ,s_{m}[L_{s}-1]\right]}^{T}$; $h_{m}={\left[h_{m}[0],h_{m}[1],\cdots ,h_{m}[L_{tap}-1]\right]}^{T}$; *L*, *L*_pn_, and *L*_s_ are the total samples number of each signal frame, PN sequence, and OFDM data block, respectively.

For the sake of obtaining the transmitted signal ***g****_m_* from received signal ***r****_m_*, accurate CIR estimation is required. In DTMB system, CIR estimation is realized according to the time-domain received FH $pn_{rec-m}={\left[pn_{rec-m}[0],pn_{rec-m}[1],\cdots ,pn_{rec-m}[L_{pn}-1]\right]}^{T}$ expressed as

(6) $$pn_{rec-m}=\left[\begin{array}{cccc} pn[0] & s_{m-1}[L_{s}-1] & \cdots & s_{m-1}[L_{s}-L_{tap}+1]\\ pn[1] & pn[1] & \cdots & s_{m-1}[L_{s}-L_{tap}+2]\\ \vdots & \vdots & \ddots & \vdots \\ pn[L_{tap}-1] & pn[L_{tap}-2] & \cdots & pn[0]\\ \vdots & \vdots & \ddots & \vdots \\ pn[L_{pn}-1] & pn[L_{pn}-2] & \cdots & pn[L_{pn}-L_{tap}] \end{array}\right]h_{m}+{\tilde{n}}_{m}$$

where $\tilde{n}$ is the AWGN vector. In Equation (6), it is clear that the received FH suffered multipath effect and was polluted by the interference caused by the preceding OFDM data block. Further, we note that the priori informationof the transmitted FH is known in the DTMB system and there is an inter-block interference (IBI)-free region of size *B* = *L*_pn_ − *L*_tap_ + 1 at the end of the received FH [32]. This IBI-free region is the premise of the CIR estimation method based on the sparse feature, which is illustrated in detail in Section 4.

### 2.3. Radar Ambiguity Function

The ambiguity function (AF) for the transmitted signal *g*(*t*) is a 2-D autocorrelation function given by the following [33,34]:

(7) $$\chi_{gg}(\tau ,f_{d})=\int_{-\infty }^{\infty }g(t)g^{*}(t-\tau )\exp(-j2\pi f_{d}t)dt$$

where *τ* and *f_d_* are the time delay and frequency offset, respectively. In the radar field, AF is an effective way to analyze the illumination signal characteristics, such as detection ability, ambiguous peaks, resolution, etc. Particularly, the DTMB signal AF can be treated as a thumbtack-like shape in a specific region of interest (ROI). For example, in the case of detecting UAV target, the detecting size is usually set to 38 m/s and 3 km, which is within the unambiguous range of DTMB signal AF [35].

For PBR system application, the target range delay and Doppler frequency can be estimated by cross-correlating (matched filtering) the reference signal *r*(*t*) with surveillance signal *e*(*t*), given as

(8) $$\chi (\tau ,f_{d})=\int_{-\infty }^{\infty }e(t)r^{*}(t-\tau )\exp(-j2\pi f_{d}t)dt$$

In practice systems, there are more than one component present in the surveillance signal. It is a result of the fact that target returns are much weaker than clutters; therefore, adaptive filtering is used before matched filtering. In the processing scheme mentioned above, the reference signal is used in clutter canceling and matched filtering. The quality of the reference signal directly affects the detection ability of the PBR system, and therefore the reference signal should be purified in advance.

## 3. Synchronizations

### 3.1. Sampling Rate Synchronization

In real DTMB system, the sampling rate is not an integer multiple of the baseband symbol rate *T*_s_ = 7.56 MS/s due to the constraint of the PBR receiver, which will cause phase offset of symbols. To keep the sampling rate and baseband symbol rate consistent, a method of cascading interpolator and decimator is implemented in this paper. The resampling reference signal is given as

(9) $$r_{\mathrm{ss}-m}\left[n\right]=\sum_{l=0}^{L-1}h_{\mathrm{ID}}\left[nD-\left\lfloor \frac{nD}{I}\right\rfloor +lI\right]r_{m}\left[\left\lfloor \frac{nD}{I}\right\rfloor -l\right]$$

where *n =* [0,1,…,*N*] denotes the samples index of resampling reference signal, and *N* denotes the total samples number of a frame signal after resampling; *I* and *D* are the interpolation and decimation factor, respectively; *h*_ID_(∙) is the cascade filter shown in [36]; ⌊·⌋ is the round-down operator.

### 3.2. Symbol Synchronization

Symbol synchronization, via the perfect autocorrelation property of FH, is performed to localize the precise start index of the FFT window. Generally, this operation can be realized by calculating the sliding correlation between the resampling reference signal and the local PN sequences, given as

(10) $$R_{s}[n]=\sum_{i=0}^{L_{\mathrm{pn}}-1}pn[i]r_{\mathrm{ss}-m}^{*}[n+i]$$

where *L*_pn_ is the samples number of PN sequence.

Further, the symbol start index $\hat{n}$ can be obtained:

(11) $$\hat{n}=\arg\underset{n}{\max}\left|R_{s}\right|$$

### 3.3. Carrier Synchronization

The orthogonality between each sub-carrier helps to extract the information of the signal. When the oscillator frequency of the transmitter and receiver are inconsistent, i.e., carrier frequency offset (CFO), serious carrier interference will be caused. The specific effect of CFO is the rotation of the constellation diagram phase. The purpose of carrier synchronization is to estimate and then compensate for CFO.

In this paper, we adopt the matched filtering method to estimate the CFO. The output of the matched filter for CFO is described by the following equation:

(12) $$R_{c-m}[\xi^{\prime }]=\sum_{i=0}^{L_{\mathrm{pn}}-1}pn_{\mathrm{rec}-m}[i]pn^{*}[i]\exp(-j2\pi i\xi^{\prime })=\sum_{i=0}^{L_{\mathrm{pn}}-1}pn[i]\exp(j2\pi i\xi_{m})pn^{*}[i]\exp(-j2\pi i\xi^{\prime })$$

where $\xi^{\prime }$ is normalized Doppler frequency, and $\xi_{m}$ is the CFO of the receiver. Further, the estimated CFO $\xi^{\prime \prime }$ can be obtained by

(13) $$\xi^{\prime \prime }=\arg\underset{\xi^{\prime }}{\max}\left|R_{c-m}\right|$$

The reference signal after carrier synchronization is given by

(14) $$r_{\mathrm{cs}-m}\left[n\right]=\sum_{n=0}^{N-1}r_{\mathrm{ss}-m}\left[n\right]\exp\left(-j2\pi \frac{n\xi^{\prime \prime }}{N-L_{\mathrm{pn}}}\right)$$

## 4. CIR Estimation Based on the Low-Rank and Sparse Properties

In this section, the detailed processes of the proposed CIR estimation method based on the low-rank and sparse property are introduced.

### 4.1. Compressed Sensing (CS) Channel Model

Numerous theoretical analyses and experimental results have verified that wireless channels associated with a number of scattering environments tend to exhibit sparse structures at high signal space dimension when working at large bandwidths and/or symbol durations and/or with a large plurality of antennas [37,38]. In other words, in a number of different communication applications, i.e., in the CIR model (3), the order *L*_tap_ of the CIR may be large, but the number of active paths *K* with significant power is usually small (*K*≪*L*_tap_). This observation motivates us to derive an accurate estimation of the sparse CIR under the new framework of CS theory.

As a new signal sampling and reconstruction algorithm, CS allows for the reconstruction of large-scale signals from limited samples. Different from the conventional sampling theory, the signal acquisition and compression are combined into one step in the CS theory [39]. Meanwhile, according to the CS theory, if the large-scale signal is sparse, or it is sparse in a certain transform domain, it can be projected onto the low dimensional space by using the dictionary matrix or sparse basis, thereby reducing the signal storage space. Assume an acquired raw signal $x\in R^{Q}$ can be represented as

(15) $$x=\sum_{i=1}^{Q}\psi_{i}\theta_{i}=\Psi \theta$$

where $\Psi ={\left[\psi_{1},\psi_{2},\cdots ,\psi_{Q}\right]}^{Τ}\in R^{Q\times Q}$ is the dictionary matrix or sparse basis; $\theta ={\left[\theta_{1},\theta_{2},\cdots ,\theta_{Q}\right]}^{T}\in R^{Q}$ is the transform domain coefficient vector. The acquired signal ***x*** is called *K*-sparse in basis **Ψ** if ***θ*** has at most *K* ≤ *Q* non-zero values, i.e., ${\left\|\theta \right\|}_{0}\leq Q$, where ${\left\|\theta \right\|}_{0}\leq \left|\mathrm{supp}(\theta )\right|$, $\left|\mathrm{supp}(\theta )\right|=\{i|\theta_{i}\neq 0,i=1,\cdots ,Q\}$ is the support of ***θ***, and for a discrete set |⋅| denotes its cardinality.

Then the signal ***x*** can be reconstructed with high probability using a small number of sampled values projected onto the low dimensional space, which can be expressed as

(16) y=Φx=ΦΨθ=Θθ

where $y\in R^{P}$ is the compressed signal, which contains main information of the raw signal; **Φ** of size *P* × *Q* (*P* ≤ *Q*) is the measurement matrix, which is used to reduce the dimension of ***x***; **Θ** = **Φ** × **Ψ** is the sensing matrix, which usually needs to satisfy the restricted isometric property (RIP) conditions.

Note that the DTMB signal purification in urban areas is considered in this paper. Firstly, in an urban environment, the actual channel length *L*_tap_ is usually much smaller than the inserted PN sequence length *L*_pn_ [38]. This means that even though the received PN sequence will be contaminated by the preceding OFDM data, there exists an IBI-free region of length *B* = *L*_pn_ − *L*_tap_ + 1 immune from the IBI at the postamble of the received PN sequence. Further, the number of resolvable propagation path *K* in an urban environment-occupied major power is relatively small (*K*≪*L*_tap_). Therefore, the IBI-free region of the received PN sequence is used to recover CIR in the DTMB system based on CS technology. Specifically, we set the IBI-free region of the received PN sequences with length *B* as the compressed signal, i.e., ***y*** = [*pn*_cs-*m*_[*L*_tap_−1], *pn*_cs-*m*_[*L*_tap_], ⋯, *pn*_cs-*m*_[*L*_pn_−1]]*^T^*, in which ***pn***_cs-*m*_ is the PN sequence in the synchronized reference signal ***r***_cs-*m*_; the sensing matrix **Θ** can be constructed by using the linear shift of the local PN sequences ***pn*** as a row vector, which can be represented as

(17) $$\Theta =\left[\begin{array}{cccc} pn[L_{tap}-1] & \cdots & pn[1] & pn[0]\\ pn[L_{tap}] & \cdots & pn[2] & pn[1]\\ \vdots & \ddots & \vdots & \vdots \\ pn_{loc}[L_{pn}-1] & \cdots & pn[L_{pn}-1] & pn[L_{pn}-L_{tap}] \end{array}\right]$$

Solving the following optimization problem, we can obtain the sparse CIR:

(18) $$\min\;{\left\|\theta \right\|}_{0},\;s.t.\;y=\Theta \theta$$

where ***θ*** denotes the sparse CIR ***h****_m_* to be solved.

Generally, to solve the optimization problem (Equation (18)), there exist two changes in the current stage. The first one is that in conventional CS theory, to obtain the accurate recovery of the original signal, the sensing matrix is usually optimized, or the CS signal recovery algorithms are modified, but the noise effect on compressed signal ***y*** is not considered. Second, among the current CS signal recovery algorithms, such as OMP, its implementation requires the sparsity level of the signal, which is variable and unknown in practical systems. The following two sections address those issues based on the DTMB inherent feature.

### 4.2. SVD Based on Low-Rank Property

In the practical DTMB system, the noise component is inevitably included in the received reference signal. Its existence will degrade the CIR estimation accuracy and thus deteriorate the target detection ability. Therefore, the noise in the reference channel should be reduced.

At present, most commercial digital illuminators and non-cooperative radars transmit signals have an inherent low-rank property [40]. For the illuminator with amplitude or phase modulation, the transmitted signal contains a periodic repetition of the same content several times in a transmitting interval, which is used to aid communication. Similarly, in the DTMB system, the transmitted signals also have a periodic repetition waveform due to the fixed PN sequence with 4 quadrature amplitude modulation (4-QAM) repeatedly inserted into the frame header, thereby giving rise to low-rank signal subspace. According to exploiting this low-rank structure, the singular value decomposition (SVD) is utilized to decrease the noise effect in the following CIR estimation.

For the sake of using the periodic repetition structure of DTMB, the reference signal within one coherent processing interval (CPI) is reshaped to multiple frames according to the symbol synchronization result firstly. Then the inserted PN sequences (FHs) are divided from these frames. Finally, a combined matrix can be obtained by stacking the divided FHs:

(19) $$PN_{\mathrm{cs}}={\left[pn_{\mathrm{cs}-1};pn_{\mathrm{cs}-2};\cdots ;pn_{\mathrm{cs}-M}\right]}^{T}$$

where ***pn***_cs-*m*_ is the received PN sequence in ***r***_cs-*m*_.

The SVD-based noise reduction method in the DTMB system is composed of three steps:

(1) Applying SVD for matrix ***PN***_cs_, Equation (19) can be expressed as
  (20) $$PN_{\mathrm{cs}}=U\Sigma V^{H}$$
  where ***U*** and ***V*** are the *L*_pn_ × *L*_pn_ right and *M* × *M* left singular matrix, respectively; $\Sigma =[diag(\sigma_{1},\sigma_{2},\cdots ,\sigma_{M}),0]$ is a *L*_pn_ × *M* diagonal matrix, where $\sigma_{m}$ is the singular value, and **0** is the null matrix.
(2) Exploiting the potential low-rank property of DTMB signal, the optimal approximation matrix of ***PN***_cs_ can be calculated by performing the inverse operation of SVD, which is given as
  (21) $$PN_{\mathrm{svd}}=U\Sigma C_{o}V^{H}$$
  where ***C****_o_* = [*I_o_*,**0**]*^T^* is a *L*_pn_ × *M* matrix, ***I****_o_* is a *o* × *o* unit matrix, and *o* is the dominant singular value number, which is set to 1 in the DTMB system.
(3) Splitting the matrix ***PN***_svd_, we can obtain the noise-reduced PN sequences ***pn***_svd-*m*_. Consequently, the IBI-free region of ***pn***_svd-*m*_ with length *B* is treated as the compressed signal, i.e., ***y*** = [*pn*_svd-*m*_[*L*_tap_−1], *pn*_svd-*m*_[*L*_tap_], …, *pn*_svd-*m*_[*L*_pn_ − 1]]*^T^*.

**Figure 3:**
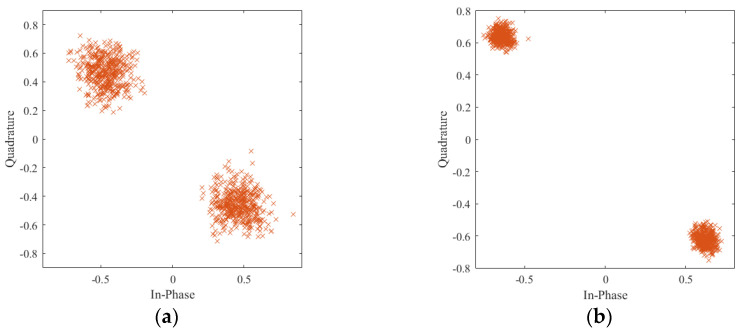
Symbol constellation diagram of measured DTMB signal without phase rotation: (**a**) before noise reduction; (**b**) after noise reduction.

**Figure 4:**
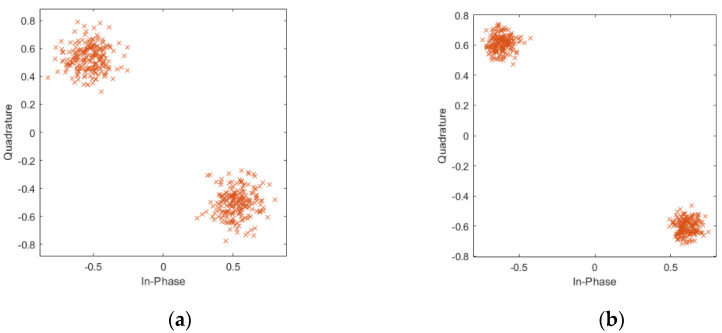
Symbol constellation diagram of measured DTMB signal without phase rotation: (**a**) before noise reduction; (**b**) after noise reduction.

Compared with signal matrix ***PN***_cs_, the noise component in the ***PN***_svd_ has been greatly compressed. Figure 3a,b depict the PN sequences constellation diagram before and after noise reduction. It is noticeable in Figure 3b that the constellation diagram becomes more clustered, which means the SNR increases. Note that, in fact, the frame header with phase rotation is also used in the DTMB system. Therefore, when the frame header with phase rotation is considered, the dominant singular value number *o* is set to 113, which is also makes sense in noise reduction, as shown in Figure 4.

### 4.3. TC-AOMP Algorithm

For the sake of obtaining an accurate signal recovery, there are a number of algorithms that have been proposed based on CS theory. Among the currently available CS signal recovery algorithms, OMP is widely adopted in the practical system due to its high reconstruction accuracy and fast convergence. The conventional OMP algorithm is to screen out the optimal-matching atoms iteratively; the signal can be recovered until the *K* atoms are selected, i.e., *K* serves as the halting criterion, where *K* denotes the non-zero values (sparsity level) of the CIR [41]. Therefore, *K* plays a crucial role in the reconstruction performance of OMP. However, *K* is generally variable and unknown in practical systems. The important CIR component will be missed if *K* is set relatively small, or the noise portion will be introduced into the estimated CIR if *K* is set relatively large. Both of these conditions will deteriorate the performance of sparse CIR estimation. Therefore, it is necessary to design a suitable halting criterion.

In this section, by fully exploiting the distinct self-correlation feature of the DTMB signal, we propose a novel TC-AOMP method to solve the optimization problem (Equation (18)). As mentioned in Section 2.3, the AF of the DTMB signal shows the thumbtack-like feature in a specific ROI. However, a series of sidelobes occur in the DTMB signal AF in the case that the received reference signal suffers the multipath effect. Inspired by this, the integrated sidelobe ratio (ISLR) is introduced in this paper. This parameter is defined as the ratio of the mean power of all sidelobes contained in a specific ROI to the power in mainlobe, expressed as

(22) $$ISLR=20\mathrm{lg}\frac{E_{\mathrm{main}}}{E_{\mathrm{side}}}=20\mathrm{lg}\frac{\Xi [0]}{\frac{1}{L_{ROI}}\sum_{l=1}^{L_{ROI}}\Xi [l]}=20\mathrm{lg}\frac{L_{ROI}\sum_{n=0}^{N-1}r_{\mathrm{eq}-m}(n)r_{\mathrm{req}-m}^{*}(n)}{\sum_{l=1}^{L_{ROI}}\sum_{n=0}^{N-1}r_{\mathrm{eq}-m}(n)r_{\mathrm{req}-m}^{*}(n+l)}$$

where *E*_main_ and *E*_side_ are the energy of mainlobe and sidelobe, respectively; Ξ[∙] is the TC result of the equalized reference signal; *L*_ROI_ is the detection range cell numbers, i.e., the size of ROI; ***r***_eq-*m*_ is the equalized signal based on the estimated sparse CIR.

**Figure 5:**
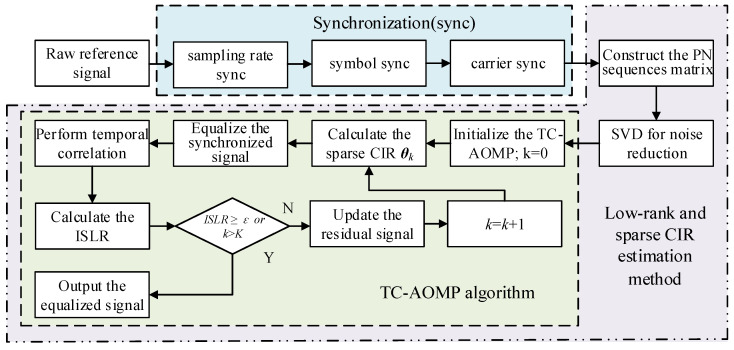
Flow chart of the proposed method.

We focus on a specific detecting range region, where the reference signal TC result shows the standard thumbtack characteristic in theory. Therefore, the ISLR provides an indication about the multipath effect on the reference signal, i.e., the ISLR can be regarded as a halting condition for judgment of whether the sparse reconstruction has reached a steady-state solution. The larger the ISLR, the better the CIR estimation performance. Finally, when the ISLR reaches the desired value, i.e., the iteration halting threshold *ε*, the optimal sparse CIR estimation can be obtained. Particularly, in the DTMB-based PBR system, the threshold *ε* can be obtained according to the noise floor of the TC result of the received reference signal. The summarized framework of the TC-AOMP algorithm is given in Algorithm 1, while all the purification processes of the reference signal proposed in this paper are shown in Figure 5. Note that, since the demodulation error rate of a communication system is typically low under normal operating conditions [42], the proposed method considers that the PBR system only performs synchronization, CIR estimation, and equalization operations, which is more efficient and suitable for real systems.
**Algorithm 1.** Summarize: The main procedures of the TC-AOMP algorithm.**0****Parameter specification: *w****_k_* is the residual; the iteration number *k*; ∅ denotes the empty set; $\Lambda_{k}$ is the index set in *k* iterations; *z_k_* is the selected index in *k* iterations; $\Omega_{k}$ is the optimal atomic set selected from the sensing matrix **Θ**; the iteration termination threshold *ε*; ***θ*** is the estimation CIR; <∙,∙> denotes the inner product operator;**1****Initialization:** set the residual ***w***_0_ = ***y***; *k* = 1; the total iteration number *K* = *L*_pre_ (*L*_pre_ is preamble length of PN sequence) $\Omega_{0}=\emptyset$; $\Lambda_{0}=\emptyset$; $\theta_{0}=\emptyset$;**2****Optimal sparse CIR estimation:** Go through each *k* in [1 *K*]with interval 1;**3****for***k* = 1,⋯,*K* **do** Calculate the inner product of the sensing matrix **Θ** and ***w***_0_, and then find the index corresponding to the maximum inner product value, which given as 
$z_{k}=\arg\underset{l}{\max}\left|<\Theta_{l},w_{k-1}>\right|,\;l=1,2,\cdots ,L_{tap},\;l\neq z_{k-1}$;**4**Update the index set $\Lambda_{k}=\Lambda_{k-1}\cup z_{k}$ and the atomic matrix $\Omega_{k}=\Omega_{k-1}\cup \Theta_{z_{k}}$;**5**Calculate the CIR via least squares at *k* iterations, as $\theta_{k}(\Lambda_{k})={\left(\Omega_{k}^{H}\Omega_{k}\right)}^{-1}\Omega_{k}^{H}y$;**6**Equalize the signal ***r***_cs-*m*_ via the estimated CIR $\theta_{k}$, obtain the equalized signal ***r***_eq-*m*_;**7**Perform temporal correlation of the equalized signal ***r***_eq-*m*_, and then calculate its ISLR;**8**If ISLR≥ε or *k* > *K*, jump out of the loop, output the equalized signal ***r***_eq-*m*_; otherwise continue iteration;**9**Update the residual $w_{k}=y-\Omega_{k}\theta_{k}(\Lambda_{k})$;**10****End**

## 5. Simulation Results

In this section, numerical simulations are provided to demonstrate the effectiveness of the proposed purification method. In order to carry out the comparison experiments, several popular CIR estimation methods, i.e., PN correlation, RLS, and conventional OMP methods, are presented. Further, the mean square error (MSE) and symbol error rate (SER) performance is also evaluated. The main simulation parameters of the DTMB-based PBR system are shown in Table 1, which are based on the Chinese DTMB standard. Two typical urban broadcasting channel models were chosen, i.e., Brazil A and COST207, to emulate the wireless channel in the practical system. The power delay parameters of Brazil A and COST207 are shown in Table 2, from which we note that the sparsity level of both channels is 6. It is assumed that each multipath is subject to an independent Rayleigh fading, and overall multipath SNR losses are normalized. Moreover, the reference signal was precisely synchronized before channel estimation.

### 5.1. CIR Estimation Results

**Figure 6:**
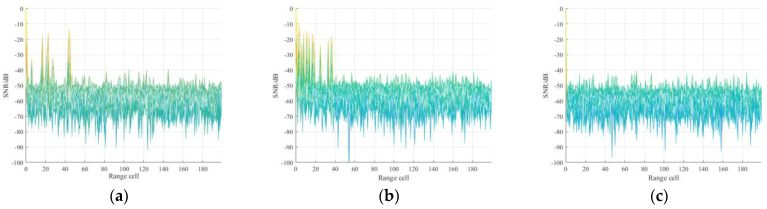
Autocorrelation results of reference signal in different channel model: (**a**) reference signal in Brazil A; (**b**) reference signal in COST207; (**c**) ideal reference signal.

In this simulation, CIR estimation ability for the two typical channel models via the proposed method is analyzed. Firstly, the guard interval length of *L*_pn_ = 420 is adopted due to the FH mode is PN420. The tap number *L*_tap_ is set to 330, which is enough to combat the urban multipath cases, so the size of the IBI-free region is 90. The SNR of reference signal is set at 10 dB. The TC results of the received reference signal are shown in Figure 6, where the reference signal in Brazil A, COST207 channel model and the ideal reference signal are shown in Figure 6a–c, respectively. It is observed from Figure 6a,b that serious range sidelobes occur due to the multipath effect and the noise floor raise compared with Figure 6c. Based on this, the ISLR is defined and used as the CIR estimation criterion in this paper. Then, for comparison, four methods, i.e., the proposed method, PN correlation, conventional OMP, and RLS, were also performed to estimate Brazil A and COST207 CIR. The CIR tap for PN correlation and RLS methods was set to 64; the iteration termination threshold for conventional OMP was set to 0.01. For Brazil A and COST207 channels, the iteration termination thresholds were set to −50 dB and −48 dB in the proposed method, respectively. The thresholds were adaptively obtained according to the TC results of the reference signal.

**Figure 7:**
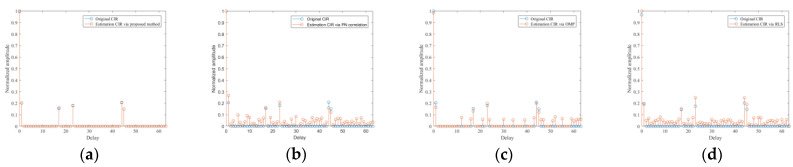
CIR estimation results in Brazil A channel of different method: (**a**) proposed method; (**b**) PN correlation method; (**c**) OMP method; (**d**) RLS method.

**Figure 8:**
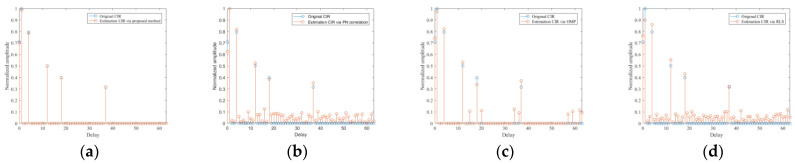
CIR estimation results in COST207 channel model of different method: (**a**) proposed method; (**b**) PN correlation method; (**c**) OMP method; (**d**) RLS method.

**Figure 9:**
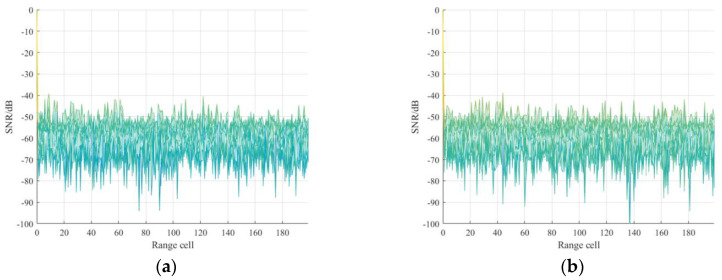
Correlation results of the equalized reference signal in different channel models: (**a**) estimated reference signal in Brazil A; (**b**) estimated reference signal in COST207.

Brazil A channel estimation results of the four methods above are shown in Figure 7. In particular, Figure 7a shows the CIR estimation result of the proposed method. It can be seen that a very exact CIR estimation result, compared with the actual channel, can be produced. With the purpose of contrast, Figure 7b shows the estimation CIR of PN correlation method and it contains many false estimations due to the mutual interference between channels and the influence of noise. Figure 7c gives the estimation CIR of conventional OMP, from which we can find that the results introduced extra tap coefficients because of the fixed number of iterations. Figure 7d shows the result of RLS; it can be seen that the estimation CIR contains lots of noise portion since the original CIR is sparse. The COST207 channel estimation results of the aforementioned methods are given in Figure 8. Specifically, Figure 8a shows that the proposed method can produce precise CIR estimation. In order to make a comparison, Figure 8b shows the result of the PN correlation method, which cannot achieve the desired CIR due to the decrease of the PN autocorrelation property caused by the mutual interference between channels and the influence of noise. Figure 8c shows the estimation CIR of conventional OMP. Since the iteration time is fixed, the result contains many false CIR estimations. Figure 8d gives the estimation results of the RLS method, in which many false taps occur because of the sparsity of the original CIR model. Consequently, the simulation results verify the superiority of the proposed method, which greatly facilitate the following signal processing. Besides, the correlation results of the equalized reference signal via the proposed method are given in Figure 9. It can be seen that the range sidelobes are suppressed greatly; meanwhile, the noise floor decreases. 

### 5.2. CIR Estimation Performance Analysis

**Figure 10:**
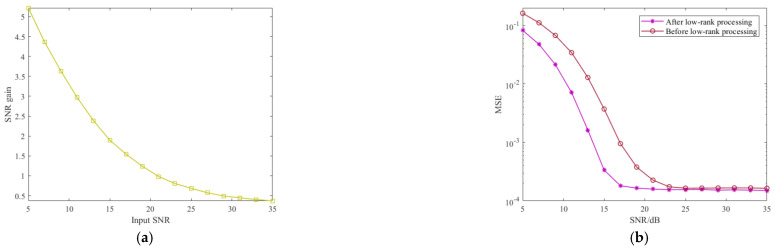
The impact of low-rank feature on SNR improvement of PN signal and CIR estimation: (**a**) SNR improvement of PN signal; (**b**) MSE performance of CIR estimation.

In this section, in order to verify the superiority of the low-rank feature in the SNR improvement of PN signal, the proposed method is conducted with different input SNR. The input SNRs vary from 5 to 35 dB. The relationship between the SNR gains of the PN signal and input SNR is given in Figure 10a. It can be seen that for different input SNR, the proposed method can obtain corresponding SNR gains based on the low-rank feature. Notably, the maximum SNR gain is greater than 5 dB in low-input SNR, which is an advantage for CIR estimation. Then, the impact of low-rank characteristics on CIR estimation performance is evaluated by MSE. The MSE performance of the proposed TC-AOMP CIR estimation method with and without the low-rank processing is performed in different SNRs, respectively. The results are given in Figure 10b, from which we can note that after the low-rank processing, the CIR estimation capacity is greatly improved under the condition of low SNR. Note that the COST207 channel model is used in this simulation.

**Figure 11:**
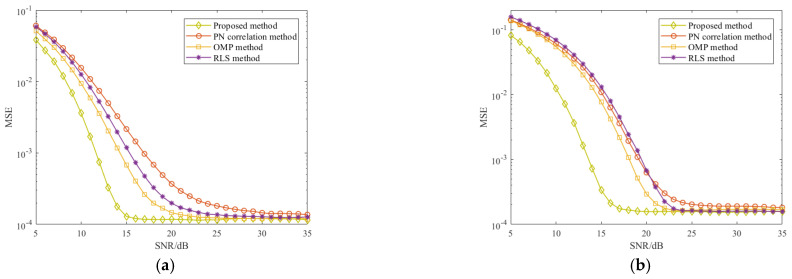

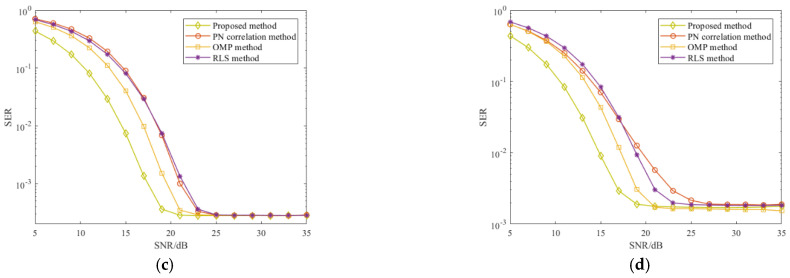
MSE and SER performance of four CIR estimation methods in different channel: (**a**) MSE performance in Brazil A; (**b**) MSE performance in COST207; (**c**) SER performance in Brazil A; (**d**) SER performance in COST207.

Finally, we compared the MSE and SER performance of the proposed method with its counterparts PN correlation, conventional OMP, and RLS methods for Brazil A and COST207 channel estimation via the Monte Carlo trials. It is worth noting that the simulation signal parameters in Table 1 were used and Gaussian noise was added to the reference signal. Figure 11 shows the MSE and SER of the abovementioned methods in different SNRs. In Figure 11a,b, it is clear that the proposed method precedes the other three methods in the case of low SNR, and with the SNR increase, the MSE performance of all methods tends to stabilize. Meanwhile, it can be seen from Figure 11c,d that the proposed method offers improvement of the SNR performance in both Brazil A and COST207 channels. Remarkably, the proposed method presents its superiority in the case of low SNR compared with the other methods.

## 6. Field Experimental Results

**Figure 12:**
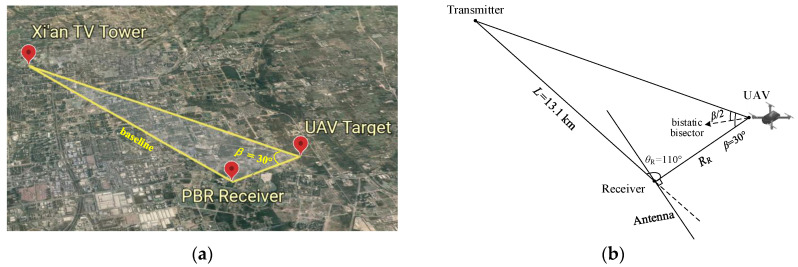
Geometry of the experiment: (**a**) the real geometric model; (**b**) the analytical model.

In this section, the field experimental data collected from a DTMB-based PBR system is used to demonstrate the purification performance of the proposed method. Figure 12 shows the geometry of the experiment, in which Figure 12a,b show the real geometric model and the analytical model, respectively. The experiment was performed on 20 July 2020, near an open space at Xidian University. Xi’an television tower was the transmitter, which is based on TDS-OFDM modulation, and the polarization method was vertical emission with the power of 1 kW. The detailed system parameters are shown in Table 1. The baseline *L*, bistatic angle *β* and the look angle of the receiver *θ_R_* were about 13.1 km, 30°, and 110°, respectively, *R_R_* was the distance between the target and receiver. The receiving antenna was a uniform linear array with eight elements. To improve the signal power, nine beams were formed by weighting the uniform linear array to receive reference and surveillance signals, where the beam coverage area was a sector varied from −60° to 60°. The receiving antenna was also vertically polarized, and the CPI was 0.2 s. The aim of this experiment was to examine the performance of the proposed method in detecting the weak target. Further, the UAV (DJI INSPIRE 2) with RCS σ = 0.1 m^2^ was used as the detection target in this field experiment, and the UAV flies away from the receiver along the normal direction of the antenna with a velocity 20 m/s and an altitude of 100 m.

**Figure 13:**
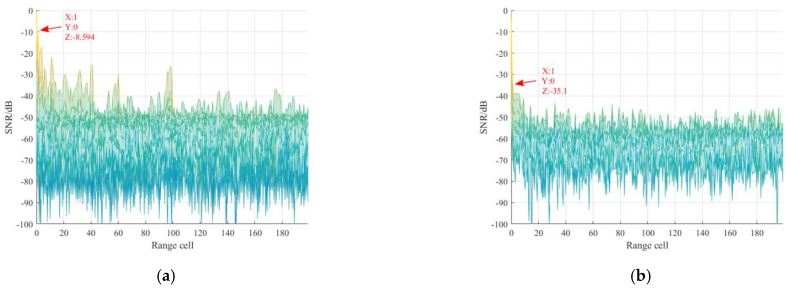
Correlation results: (**a**) reference signal before purification; (**b**) reference signal after purification.

**Figure 14:**
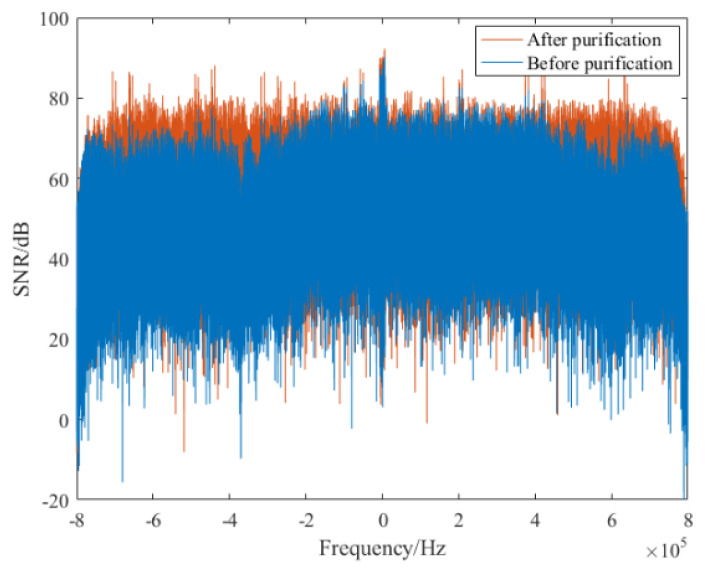
Spectrum comparison of reference signal before and the after purification.

**Figure 15:**
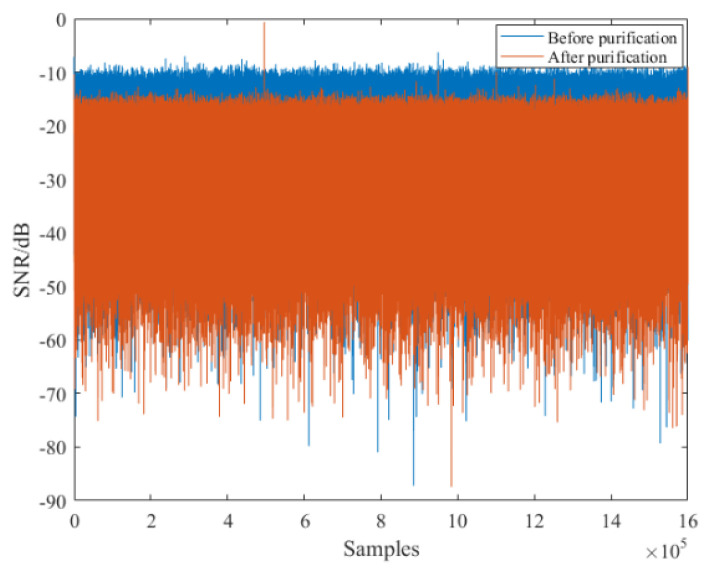
Clutter suppression result comparison of reference signal before and the after purification.

After the synchronization operation of the received reference signal, its TC result is described in Figure 13a, from which we can find that serious range sidelobes occur due to the multipath effect, and the power of maximum range sidelobe is −8.594 dB, as marked in the figure. Then, the purified reference signal was obtained via the proposed method, and its TC result is given in Figure 13b. It can be seen from this figure that all range sidelobes are suppressed greatly. In particular, the first sidelobe is decreased to −35.1 dB, and we can clearly note the noise floor is also decreased. Besides, the spectrum comparison results between the reference signal before and the after purification are given in Figure 14. It is noted that the purified reference signal spectrum becomes smoother compared with the original spectrum. These measurements show that the multipath effect in the received reference signal has been sufficiently reduced. After the purification operation, the purified reference signal is used for clutter suppression. In order to make a comparison, the reference signal before purification is also utilized for clutter suppression. The clutter suppression comparison results are given in Figure 15. It is noted that after the purification, the clutter suppression result was reduced by more than 5 dB. In order to fully demonstrate the performance of the proposed method, the clutter suppression of different reference signal purification methods was also performed. The clutter elimination ratios of different purification methods are given in Table 3. It is worth noting that the clutter suppression performance of the proposed method exceeds the PN correlation, OMP, and RLS methods, which contributes to improving the target SNR. Note that the clutter ratio of reference signal without purification is 5.4 dB.

**Figure 16:**
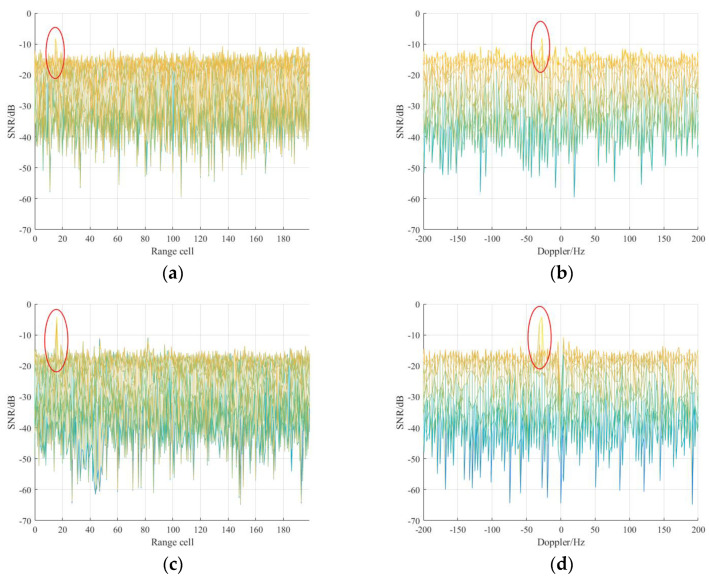

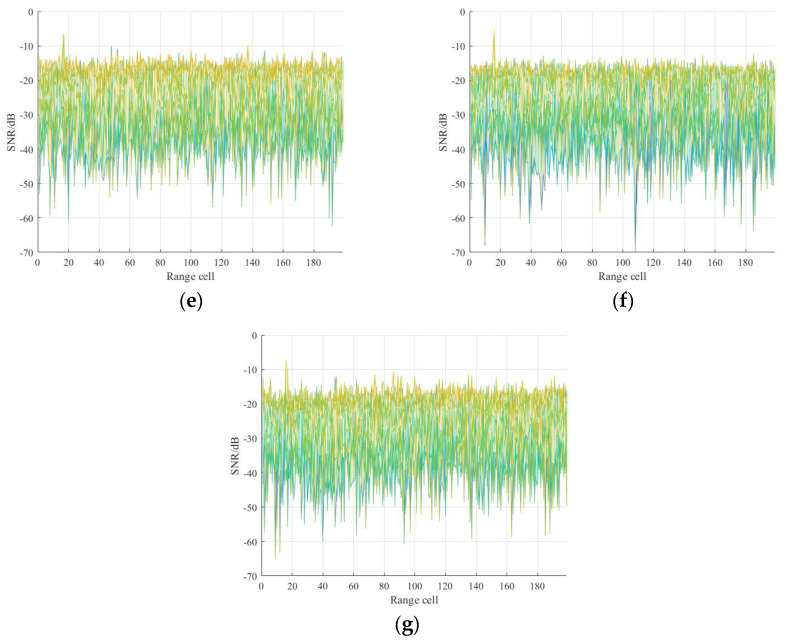
Target detection results of measured data using different purification method: (**a**) reference signal before purification in the range dimension; (**b**) reference signal before purification in the Doppler dimension; (**c**) the proposed method in the range dimension; (**d**) the proposed method in the Doppler dimension; (**e**) PN correlation method; (**f**) OMP method; (**g**) RLS method.

Finally, range-Doppler processing was performed to detect the UAV target, and the integrated results of these methods are represented in Figure 16. It can be observed that all the purification methods can improve the target SNR compared with the reference signal without purification. More specifically, among these purification methods, the proposed method has the largest increase in SNR, about 5 dB, which makes target detection easier, and the real range *R_R_* of UAV target is 1.52 km according to the real geometry.

## 7. Conclusions

In this paper, a novel sparse CIR estimation scheme for reference signal purification in DTMB-based PBR systems is proposed. By leveraging the low-rank structure of DTMB signal and the sparsity feature of wireless channels, the proposed method can acquire reliable channel estimation and thus improve the target detection performance. More specifically, synchronizations, including symbol, carrier, and sampling rate synchronization, are performed to reference signal at first, and the corresponding PN sequences can be acquired. After that, by exploiting the inherent low-rank structure of DTMB signals, the noise component in PN sequences is suppressed. Further, the TC-AOMP method is proposed to estimate the sparse CIR, in which a new halting criterion is defined based on the TC result to further improve the channel estimation accuracy. In the end, the purification reference signal is obtained via equalization operation. Compared with the existing methods, the proposed method can obtain superior CIR estimation performance, especially in low SNR conditions. Simulated and experimental results were carried out to verify the effectiveness of the proposed method in detail. Although the proposed method can achieve satisfactory performance in the DTMB-based PBR system, the large and time-varying delay spread (greater than GI) is often considered in the PBR system as well. Combining the DTMB-based PBR feature with the more complicated multipath effect is an interesting topic and will be part of our future study.

## Figures and Tables

**Table 1:** Parameters of PBR system.

Description	Parameter	Value
Total subcarriers	*K*	3780
Carrier frequency	*f_c_*	666 MHz
Sample frequency	*f_s_*	8 MHz
Bandwidth	*B*	8 MHz
polarization mode	*-*	vertical
Power	*-*	1 kW
Carrier spacing	∆*f*	2 kHz
Sample rate	1/*T*_s_	7.56 MSPS
Signal constellation	*-*	16 QAM
Frame header mode	*-*	PN420

**Table 2:** Parameters of channel models.

Description	Brazil A						COST207					
Tap	1	2	3	4	5	6	1	2	3	4	5	6
Delay (μs)	0	0.15	2.22	3.05	5.86	5.93	0	0.2	0.6	1.6	2.4	5.0
Power (−dB)	0	13.8	16.2	14.9	13.6	16.4	3	0	2	6	8	10

**Table 3:** Clutter suppression ratio of different purification method.

Method	PN Correlation	OMP	RLS	Proposed Method
Clutter suppression ratio	7.8 dB	8.7 dB	8.1 dB	10.6 dB
